# “It's something I'll do until I die”: A qualitative examination into why older women in the U.S. continue screening mammography

**DOI:** 10.1002/cam4.4758

**Published:** 2022-05-26

**Authors:** Laura E. Brotzman, Rachel C. Shelton, Jessica D. Austin, Carmen B. Rodriguez, Mariangela Agovino, Nathalie Moise, Parisa Tehranifar

**Affiliations:** ^1^ Department of Sociomedical Sciences Columbia University Mailman School of Public Health New York New York USA; ^2^ Herbert Irving Comprehensive Cancer Center Columbia University Medical Center New York New York USA; ^3^ Department of Epidemiology Columbia University Mailman School of Public Health New York New York USA; ^4^ Department of Medicine Columbia University Irving Medical Center New York New York USA

**Keywords:** aging, breast cancer, de‐implementation, overscreening, qualitative, screening mammography

## Abstract

**Background:**

Professional guidelines in the U.S. do not recommend routine screening mammography for women ≥75 years with limited life expectancy and/or poor health. Yet, routine mammography remains widely used in older women. We examined older women's experiences, beliefs, and opinions about screening mammography in relation to aging and health.

**Methods:**

We performed thematic analysis of transcribed semi‐structured interviews with 19 women who had a recent screening visit at a mammography clinic in New York City (average age: 75 years, 63% Hispanic, 53% ≤high school education).

**Results:**

Three main themes emerged: (1) older women typically perceive mammograms as a positive, beneficial, and routine component of care; (2) participation in routine mammography is reinforced by factors at interpersonal, provider, and healthcare system levels; and (3) older women do not endorse discontinuation of screening mammography due to advancing age or poor health, but some may be receptive to reducing screening frequency. Only a few older women reported having discussed mammography cessation or the potential harms of screening with their providers. A few women reported they would insist on receiving mammography even without a provider recommendation.

**Conclusions:**

Older women's positive experiences and views, as well as multilevel and frequently automated cues toward mammography are important drivers of routine screening in older women. These findings suggest a need for synergistic patient, provider, and system level strategies to reduce mammography overuse in older women.

## Graphical abstract

Older women’s positive experiences and views towards mammography, as well as multilevel and frequently automated cues for mammography are important drivers of routine screening. These findings suggest a need for synergistic patient, provider, and system level strategies to reduce mammography overuse in older women.

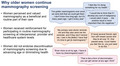

## 1 INTRODUCTION

Screening mammography remains the cornerstone of population‐level efforts to reduce morbidity and mortality from breast cancer, but also poses potential burden and harms including treatment of screen‐detected breast tumors that would not have affected women's health and mortality (commonly referred to as overdetection and overtreatment).^1^, ^2^ As randomized control trials and most organized population‐level screening programs have not included women ≥75 years, the evidence on the tradeoffs between benefits and harms of mammography in older women is limited. The decreasing life expectancy and increasing risks of comorbidities and functional limitations associated with aging suggest lower benefits from mammography for breast cancer mortality reduction in older women.^3^, ^4^, ^5^

The US Preventive Services Task Force (USPSTF) does not make breast cancer screening recommendations for women ≥75 years citing insufficient evidence,^6^ while the American Cancer Society recommends screening for women with a life expectancy of 10 years or more,^7^ and the American College of Physicians supports discontinuing mammography in women ≥75 years at average risk for breast cancer.^8^ Despite some variations in how best to approach screening mammography among older women, all U.S. professional guidelines support individualized and informed decision‐making, including a balanced discussion of screening benefits and harms, and consideration of clinical risk factors and patient preferences. In contrast to many countries that provide breast cancer screening through organized programs, women in the U.S. access mammography through referrals by healthcare providers, and to a lesser extent, through self‐referral within healthcare systems, making the implementation of the screening guidelines variable across healthcare settings and populations.^9^ Recent national data show that nearly three‐quarters of women in the U.S. report having at least one screening mammogram at ages 75 and older, and over half report having had a mammogram within the past 2 years.^10^, ^11^ Evidence suggests that screening may decline with increasing age and decreasing life expectancy, yet as many of 36% of older community dwelling women and 56% of older breast cancer survivors with less than 5 years life expectancy report having received a mammogram in the past 1–2 years.^12^, ^13^ These data are consistent with research showing that most providers endorse and continue to recommend mammography to their older female patients, and many are hesitant to recommend against breast cancer screening, due in part to feeling ill‐prepared to discuss life expectancy and mammography cessation, and anticipating negative reactions from patients.^3^, ^14^, ^15^, ^16^

Reducing overuse of screening mammography in older women is further complicated by uncertainties around estimating life expectancy, healthcare incentives for providing mammography services regardless of age and health considerations, and public health campaigns and popular support for breast cancer screening.^17^, ^18^ Limited available research suggests that screening mammography among older women may be habitual and perceived as reassuring and easily accessible.^12^, ^15^, ^19^, ^20^, ^21^ However, very little is known about older women's perspectives and experiences with regard to reducing or stopping mammography, particularly among racial or ethnic minority women in the US or samples from racially and ethnically diverse populations.^21^ To address this gap, this study seeks to provide an in‐depth understanding of the perspectives and preferences of older, ethnically diverse women as they relate to continued screening and potential overuse of routine breast cancer screening.

## 2 METHODS

### 2.1 Recruitment and data collection

As part of a pilot study to understand factors contributing to screening mammography in older women, we recruited 52 women ages 70 years and older as they presented for their screening mammography appointment. Recruitment was conducted at a large mammography clinic in New York City that serves a racially and ethnically diverse and predominantly low‐income and immigrant population (>75% Hispanic, Spanish‐speaking women) with high rates of routine screening mammography (87.9%). We selected this recruitment approach to ensure diverse representation of older women who actively receive screening mammography and included women 70–74 years to provide insight into the experiences of women who were approaching the age for which screening guidelines change. Participants completed an in‐person survey, administered by bilingual (English and Spanish) research staff at the time of mammography appointment, to collect data on sociodemographic, breast cancer risk factor, clinical and screening mammography history.^22^ All participants consented to be contacted for follow‐up studies, and were later invited to participate in semi‐structured interviews to collect in‐depth data on women's perspectives on reducing or stopping routine screening mammography. Two members of the research team (one Bilingual English‐Spanish‐speaking, one English‐speaking) conducted all interviews, lasting between 30 and 45 min, between June and August 2019; these data serve as the focus of the current study. Interviews were audio‐recorded and transcribed verbatim in their original language, and transcribed Spanish interviews were subsequently translated to English. Recruitment for semi‐structured interviews continued until no new themes were identified and thematic saturation was reached.^23^, ^24^ Women completing the interviews were representative of the overall sample but were less likely to be foreign born (68% vs. 81%) and less likely to be primarily Spanish speaking (63% vs. 74%).

The study was approved by the Columbia University Medical Center Institutional Review Board. All participants provided written informed consent at the time of enrollment into the study and additionally provided verbal informed consent before completing semi‐structured interviews.

### 2.2 Interview guide

Broadly informed by national recommendations for patient‐provider discussions and literature regarding informed decision‐making processes for breast cancer screening,^7^, ^8^, ^25^, ^26^, ^27^ we developed an interview guide to elicit older women's experiences, beliefs and intentions about screening mammography. Specifically, women were asked about the process of receiving mammography, pros and cons of screening mammography, and what influenced their screening decision (see supplemental material). We also presented participants with two vignettes of 78‐year‐old women with no family or personal history of breast cancer but with differing health status to obtain information about women's reaction and understanding of age and health considerations in screening mammography recommendation. Specifically, the first vignette described the woman as being in good health while the other described the woman as having diabetes and difficulty handling her day‐to‐day activities on her own. The vignettes also state that the depicted woman's provider recommended reducing or discontinuing screening mammography due to age (both vignettes) and poor health (second vignette only), but left the choice up to the patient. After presenting each vignette, participants were asked if they thought either woman should receive a mammogram, and why or why not.

### 2.3 Data analysis

We conducted interviews with 19 women in English (*n* = 7) or Spanish (*n* = 12) by phone (*n* = 12) or in person (*n* = 7). Two members of the research team trained in qualitative methods (LB, JA) conducted thematic analysis,^28^, ^29^ using an iterative deductive‐inductive coding and analytic approach.^30^ Each reviewer independently reviewed each transcript line‐by‐line, applying an initial list of deductive codes based on the interview questions and probes (e.g., reasons/motivations for screening, attitudes and beliefs about mammograms, screening behaviors, prompts or cues for screening, decisional influences, and vignette responses). To allow the codebook to expand and be refined, we used an inductive data‐driven approach to identify new codes, as well as themes across codes (see supplemental material for codebook).^31^ The final code structure was applied to all transcripts. To facilitate the reliability of the coding process, the two reviewers coded four transcripts together, which was followed by independently reviewing the remaining 15 transcripts before coming together to compare codes and resolve discrepancies with the larger research team. NVivo 12 software (QSR International) was used for coding to facilitate collaborative analysis, organization, and interpretation. We explored the possibility and found little support that results were different for women younger and older than 75 years.

## 3 RESULTS

### 3.1 Sample characteristics

Women ranged in age from 71 to 83 years (see Table 1). The majority of women in the sample identified as Hispanic or Latina (63%; hereafter Hispanic) and were primarily Spanish‐speaking (63%) (Table 1). Based on survey data, over half of the women had a high school degree or lower, about one third reported having had a breast biopsy, and none had a personal history of breast cancer. More than half of women rated their general health as “fair” or “poor” and reported an average of 4.4 physician diagnosed medical conditions (e.g., hypertension, diabetes). Over 68% of women reported having had annual mammogram in the past 5 years.

**TABLE 1:** Sample demographics and health characteristics (*n* = 19)

	*n* (%)/mean (*SD*)
Age (71–83 years)	74.89 (3.78)
Interview Language	
English	7 (36.8)
Spanish	12 (63.2)
Race	
Hispanic	12 (63.2)
Non‐Hispanic White	6 (31.6)
Non‐Hispanic Black	1 (5.3)
Nativity/birthplace	
US – born	6 (31.6)
Foreign – born Dominican Republic	7 (36.8)
Foreign – born Other country^a^	6 (31.6)
Education	
Less than high school	7 (36.8)
High school or GED	3 (15.8)
Some college	4 (21.1)
Bachelor's or advanced degree	5 (26.3)
Self‐rated health	
Poor	3 (15.8)
Fair	7 (36.8)
Good	3 (15.8)
Very good	6 (31.6)
Excellent	0
Number of Health Conditions^b^	4.36 (1.89)
1–2	2 (10.6)
3–4	9 (47.4)
5+	8 (42.2)
Health Literacy^c^	
Adequate	7 (36.8)
Marginal	7 (36.8)
Low	5 (26.3)
Breast cancer risk factors and screening characteristics	
First degree family history of breast cancer	3 (15.8)
History of breast biopsy	6 (31.6)
Discussed stopping mammography with PCP	2 (10.5)
Discussed stopping mammography with family	2 (10.5)

^a^ Other countries include: Cuba (1), Mexico (1), Ecuador (2), Colombia (1), Germany (1).

^b^ Self‐reported medical conditions that had been diagnosed by a doctor including diabetes, myocardial infarction, osteoporosis, high cholesterol, thyroid, etc.

^c^ Assessed using three questions developed by Chew et al. (2004).

### 3.2 Qualitative themes

We identified three overarching themes related to continued use or cessation of screening mammography as described below. Additional quotes reflecting these themes are provided in Table 2.

**TABLE 2:** Illustrative quotes by major themes

Theme	Quotes
1. Mammograms as a beneficial and routine part of care	“I'm going to be 84… Anytime they want to give me a test, I'm there. It always helps.” (Age 83, Non‐Hispanic White) “I think every woman should have it! That's just how I feel. Every woman. I do not care what else is going on. Once a year you are going for the screening. You go have it done.” (Age 71, Non‐Hispanic Black) “I would like to think that if I develop any sort of malignancy I would catch it early… the positive result is more important than the negatives” (Age 79, Non‐Hispanic White) “As long as the doctors let me do it, I'll keep doing it” (Age 71, Hispanic)
2. Participation in routine mammography screening was reinforced at multiple levels	Interpersonal level “No one's ever said ‘do not go [get a mammogram].’” (Age 71, Non‐Hispanic Black) “I've gotten mammograms ever since we were told that we could get them… My mother died at the age of 46 from breast cancer… her mother died at 94 but she had a different breast cancer at 74… That's why I go” (Age 83, Non‐Hispanic White)
	Provider level “[My doctor] recommends still going…She felt it was a good idea to continue once a year. And I decided to take her advice.” (Age 79, Non‐Hispanic White) “Everything that a doctor orders is for a reason. And we have to submit to it 100%, to any exams that the doctor order for us. That's my opinion” (Age 71, Hispanic) “I am referred – you know I am told that it's required.. and so I do it” (Age 71, Non‐Hispanic White). “I call the doctor – the gynecologist – and she gives me the referral” (Age 77, Hispanic) “My health is important to me. If he tells me that I have to go, then I have to go get checked” (Age 77, Hispanic)
	Healthcare system level “Well, my primary care doctor reminds me and they also send me the reminder [letter], and if they do not remind me, I see in the [letter] I have that it's time to get [a mammogram].” (Age 72, Hispanic) “I got a letter that says ‘your mammogram, it should be scheduled after a certain date, so there's covered by social security Medicare.’ And I made my appointment, came in, got the mammogram” (Age 75, Non‐Hispanic White) “I got the letter and I called and made the appointment” (Age 79, Non‐Hispanic White)
3. Older women did not endorse discontinuation of mammography screening due to advancing age or diminishing health	Age “It does not matter the age. We can get breast cancer at any age.” (Age 77, Hispanic) “Why is it that someone, let us say 80 years old, which is what I am, they do not want to do a pap smear anymore because they say that after a certain age they cannot do it, or whatever exam that's deeper or more thorough? Why cannot they do it at that age?” (Age 80, Hispanic) In response to vignette: “That's something I want to learn about…when they say you can stop getting mammograms. That's interesting. So I would say she better find out why he said that because of her age” (Age 75, Non‐Hispanic White). “I think about the age thing. You know, if there's a cut‐off point that tells you to go every 5 years instead of every year… I would go” (Age 74, Non‐Hispanic White)
	Health “…diabetes has nothing to do with breast cancer” (Age 72, Hispanic)
	Provider recommendation against screening “I would not do it.. I would not do it. Why would I get it if my doctor tells me not to?” (Age 78, Hispanic) “If one doctor says no [do not get a mammogram], I would say you do not have to listen to him. Listen to yourself.” (Age 75, Non‐Hispanic White)

#### 3.2.1 Older women perceived mammograms as a positive, beneficial, and routine component of their healthcare

Many women reported having a long history of receiving mammograms and perceived mammography as a necessary and routine part of their care. Most women reported receiving annual mammograms; as one woman shared: “*I've gotten mammograms ever since we were told that we could get them…I don't know how long ago, but it's many years ago. I get it every year”* (Age 83, Non‐Hispanic White). Additionally, many women expressed strong intentions to continue screening mammography as long as possible, with one woman sharing: *“It's something I will do until I die”* (Age 75, Non‐Hispanic White).

Commonly reported benefits of mammography included the early detection of cancer and initiation of treatment, and perceived reassurance of receiving results that did not detect cancer. The few negatives reported by women were limited to physical pain during the mammography procedure, concern about potential exposure to radiation, and fear of finding out they had cancer. Most women perceived that the benefits of screening mammography outweighed any of these potential negatives. One woman explained:

“I can say the upside is that it diagnoses breast cancer early enough for women to get treatment. That outweighs the pain that you have momentarily… You can't even compare the two.” (Age 71, Non‐Hispanic Black).

The majority of women described their experience of getting a mammogram as “*easy*” in many respects (e.g., procedure is not invasive, appointments are easy to make, and the screening is covered by insurance). A few women reported that they feel well cared for during their appointment, and that the screening visit provided an opportunity for social connection. One woman shared: “*Well, I think at this point I would [continue to get mammograms], because I've always done it, and you know what? I'm retired now, so I need some place to go*” (Age 83, Non‐Hispanic White).

#### 3.2.2 Older women's participation in routine screening mammography was reinforced at interpersonal, provider, and healthcare system levels

All women reported regularly receiving one or more reminders to have a mammogram. These included reminders in the form of providers' recommendation and referrals during clinical visits, healthcare system reminder letters or calls, and for a few women, their own personal annual calendar reminder. One woman recalled receiving a letter from the mammography clinic stating that “it was time for me to get it done” (Age 79, Hispanic). Another woman described multiple cues: *“I did get a [reminder] letter from the [mammogram] clinic. And then I was going for a checkup and I asked the doctor to give me a [referral] for a mammogram*” (Age 71, Non‐Hispanic White).

A few women also stated that knowing someone close in age with breast cancer reinforced the need to continue screening. One woman explained: *“[I have] several friends right now with breast cancer and they're all over 70. One passed away. It just makes you feel like no, you just need to do this [get screened]”* (Age 71, Non‐Hispanic Black). Another woman shared her family history of breast cancer later in life motivated her to continue screening into her late 70s: *“I had an aunt who had a malignancy in her 70s. It made sense to me”* (Age 79, Non‐Hispanic White).

The majority of older women indicated that their primary care provider or gynecologist typically recommended screening mammography and provided referrals during annual visits (“*My doctor always asks about my mammogram during my annual checkup*” (Age 71, Non‐Hispanic Black)), even for women older than 75 (*“[My doctor] always reminds me, he always reminds me and gives me the referral”* [Age 76, Hispanic]). Most women trusted that provider's recommendation or referral for a test meant that it was important and necessary for them to adhere to their recommendation: *“I know that when they [providers] order a test for you it's because it's necessary…every time that they tell me to, every year I get a mammogram”* (Age 77, Hispanic).

We asked women whether their providers had discussed discontinuation of screening mammography. Only one woman reported having had such discussion: “*I told the doctor that I wanted a mammogram, and she ordered one. But before she had told me that it wasn't necessary for me [to continue being screened]…because of my age”* (Age 79, Hispanic). When asked about the information they had received to inform decisions about screening mammography, no women reported receiving information from their providers about the potential harms or lower benefits of screening mammography in older ages.

#### 3.2.3 Older women did not endorse discontinuation of screening mammography due to advancing age or poor health

Overall, older women were surprised about the idea of discontinuing screening mammography in older ages, and perceived mammography to be increasingly more important as women age and their health declines. Additionally, some women expressed confusion about the idea that a woman's health conditions would impact continued receipt of mammography. One woman explained: *“Even more so at my age, I have to have my [mammogram] exam. And if the doctor recommends that I have it more, and I know that it's for my own well‐being, then I do it diligently.”* (Age 71, Hispanic).

When presented with the vignettes, the majority of women did not understand why the provider would not recommend mammography due to older age and/or health condition. Several women were confused about the role of age in screening, asking: *“Older women, like me, should get a mammogram, or no? That's the question I have now”* (Age 79, Hispanic) and *“In this scenario, at 78, she doesn't need to really continue getting mammograms. Why? Why is there a cutoff point?*… *I'll be 76 this month…So yeah, I'd be interested in knowing that” (Age 75, Non‐Hispanic White)*. Regarding health, an older woman asked: “*What does her medical condition got to do with her getting mammograms*?” (Age 79, Non‐Hispanic White).

When asked what they think the older women in the vignettes should do, some women advised that they should follow the guidance or recommendation of their provider, but a few said that the women depicted in vignettes should still consider screening regardless of provider recommendation and current health conditions, citing that their own provider continues to recommend screening mammography (see Table 2). A few women expressed that they would insist on receiving mammograms even if not recommended by their provider.

When probed about how they feel about a provider recommending against screening mammography due to advancing age and/or declining health, a few perceived it as unfair and potentially biased. As one woman explained: *“That is like a form of discrimination* … *you can get cancer at any age I think, .. I think that mammography should be done*” (Age 72, Hispanic). Of note, several women specifically reported they would feel comfortable getting mammograms less frequently (e.g., every 2–3 years), but not stopping altogether. One woman explained her rationale in the following statement: *“Until you die, your hormones are working. So it's not about stopping mammograms but getting them less frequently”* (Age 75, Hispanic). Still some women likened the discontinuation of screening mammography to no longer receiving screening for cervical cancer due to increasing age. For example: *“When they told me that I didn't have to get the pap smear anymore, I said ‘oh, that's fine’ and I never got it again*…*.. I would accept [stopping screening mammography] just like I agreed about the pap smear”* (Age 71, Hispanic).

## 4 DISCUSSION

We found that older, predominantly Hispanic women who participated in routine screening mammography reported positive experiences (e.g., social interaction, ease of receipt) and perceive the benefits of early detection and treatment to largely outweigh the few perceived negative aspects of mammography. Women's positive beliefs and the routinized nature of their participation in screening mammography were often reinforced by various factors and cues to action such as knowing someone with breast cancer, provider recommendations, and healthcare system reminders such as letters. Further, the majority of older women in this study were unaware of the potential harms of screening in terms of false positive results and overdetection, nor did they view advanced age or comorbidities as important considerations in deciding whether to receive screening mammography.

Findings reported here are consistent with prior studies suggesting screening mammography among older women is habitual and even considered as a “moral obligation”.^12^, ^15^, ^19^, ^20^ After years of participating in screening, older women in this study perceived the value of mammography for early detection of cancer, drew reassurance from mammograms with negative breast cancer findings, and believed mammography to be easily accessible and essential to monitoring their health as they get older. Despite the consideration of age, life expectancy, and health status in guideline recommendations for the discontinuation of screening mammography, older women in this sample perceived that older age and poor health underscored a need for continued screening. While these findings may reflect the low awareness of the potential harms of screening reported by women in this and other studies,^15^, ^20^, ^21^, ^22^ they also point to a possible disconnect between factors that the medical and research communities deem important for screening recommendations in older populations, and the values and concerns that influence screening mammography decisions among ethnically diverse older women.

Changing long‐standing beliefs and habitual behavior may be challenging when they are supported at multiple levels within healthcare systems and in the broader society. Older women in this study stated strong intentions to continue screening mammography, which was reinforced by interactions with their providers and system level prompts (e.g., reminder calls and letters from providers, clinics, and health insurance companies). The central importance of providers' recommendations for promoting screening mammography has been well documented for women of screening ages and for older women,^32^, ^33^ and while the women in this study believed in following their providers' recommendation, only one reported having had a discussion with her provider about benefits and harms of mammography or even the option to not screen. This is consistent with research suggesting the continuation of cancer screening among older adults is not typically characterized as a decision, but as an automatic behavior.^34^, ^35^, ^36^ Additionally, several women in this sample aged 75 and older specifically asked questions about the relationship between age and stopping screening and if they should still be screening at their age, suggesting that providers are not discussing discontinuation with women who should be targeted for these conversations. Previous research suggests that providers favor discussing the benefits and rarely discuss possible harms of screening mammography,^37^ and express discomfort and concerns about limited time and reliable tools to facilitate complex discussions about tradeoffs and cessation of screening.^15^, ^38^ These findings highlight the need to develop and test provider‐level strategies to improve individualized assessment of older women's life expectancy along with communication approaches for adequate presentation of both potential benefits and risks of screening mammography in older ages. Further, these strategies may need to be adapted to the needs of racially and ethnically diverse populations with varied experiences with providers and healthcare systems. For example, as compared to Black and White women, Hispanic women have been reported to be least confident about advocating for their own preferences regarding breast cancer screening, and reported “following doctors' orders” without question.^39^ Modifying cues such as automatically generated reminder communication from healthcare systems to instead encourage older women to discuss screening with providers may further help to support provider recommendations around screening cessation as appropriate and reduce unnecessary screening.

In this study, most women did not express a willingness to stop screening mammography, and a few noted they would continue screening mammography even if their provider no longer recommended it or refused to provide a referral. A few older women even viewed mammography cessation as a form of age discrimination. This finding is in agreement with reports that older adults are skeptical and even suspicious of recommendations to stop cancer screening.^19^, ^40^, ^41^ While concerns about mammography cessation were reported, multiple women stated an openness and interest in receiving mammograms less frequently (e.g., every 2–5 years). Among women who fear being unable to detect cancer if they stop receiving mammograms altogether, reducing the frequency of screening might be a possible strategy to address overuse of screening in this population. Emphasizing individual preferences may help alleviate potential harms and concerns caused by complete cessation while also reducing unnecessary screening. Finally, several women in this study likened the possibility of mammography cessation to their experience with stopping cervical cancer screening. Framing and normalizing discussions about stopping mammography in a similar way as recommendations to stop receiving other preventive care may be a useful way for providers to introduce and discuss cessation among this population.^42^ As such, improving provider discussions about when to stop screening by addressing women's concerns surrounding cessation are critical. Additional research on aligning these discussions with patient values and preferences in socio‐demographically diverse populations is needed.

We did not verify patient‐reported data by chart review or provider interview, and therefore cannot rule out the potential for some error in recalling prior discussions with providers. Nonetheless, the reliance on women's reports is suitable for capturing the experiences of older women with respect to screening mammography. While these findings include perspectives from women ages 70–75 for whom screening mammography is recommended, a notable strength is the inclusion of older women (ages 75–83) who may be susceptible to overuse of screening mammography and whose perspectives shed light on multilevel factors facilitating their continued screening. Our intent was to gain a fuller understanding of the experiences and views of women who are approaching as well as those past the age for which guidelines change. We explored the possibility and found little support that results were different for women younger than 75 years from older women. The qualitative design of this pilot study and small sample size limits our ability to explore differences in perspectives by meaningful sociodemographic subgroups such as race, ethnicity, or nativity. However, the ethnic composition of this sample adds to a literature lacking the perspectives of urban, older Hispanic women, and those with multiple chronic conditions and serves as an important exploratory step toward better understanding experiences and perceptions of this group. Finally, we acknowledge this study may not be generalizable to older women outside of the US where healthcare systems and screening mammography guidelines and perspectives may differ.

The research presented here provides in‐depth insights into the experiences of older women in the U.S. receiving routine screening mammography and highlights many of the challenges that must be overcome to reduce the potential overuse of screening mammography in this population. These findings suggest a need for increasing awareness of screening harms and opportunities for cessation at the patient level and facilitating patient‐provider discussions that incorporate individualized health assessment as well as patient values and preferences into screening mammography decision‐making for older women. More broadly, there may be opportunities to apply frameworks and accumulating evidence from the field of implementation science focused on “de‐implementation” of practices that are low‐value, costly, or lack an evidence base to help inform identification and development of additional strategies.^43^

## AUTHOR CONTRIBUTIONS

LB analyzed and interpreted interview data and drafted manuscript. JA analyzed and interpreted interview data and reviewed the manuscript. CR and MA coordinated data collection and management, performed interviews, and reviewed the manuscript. PT and RS developed the protocol and science. PT, RS, and NM guided data analysis and interpretation and revised the manuscript. All authors read and approved the final manuscript.

## FUNDING INFORMATION

LB received support from the National Institutes of Health (grant: T32 AG027708).

## CONFLICT OF INTEREST

The authors have no conflict of interest to declare.

## PRECIS FOR USE IN THE TABLE OF CONTENTS

Older women's positive experiences and views toward mammography, as well as multilevel and frequently automated cues for mammography are important drivers of routine screening. Findings suggest a need for patient, provider, and system level strategies to reduce mammography overuse in older women.

## ETHICS STATEMENT

The study was approved by the Columbia University Medical Center Institutional Review Board. All participants provided written informed consent.

## Supporting information


Data S1

## Data Availability

The author elects to not share data.
