# Supplementary material for: “It's something I'll do until I die”: A qualitative examination into why older women in the U.S. continue screening mammography
Source: Cancer Med. 2022 May 26;11(20):3854–62. doi: 10.1002/cam4.4758 (PMC9582674; doi:10.1002/cam4.4758)
Supplement: Supplementary file 1 — Data S1 [file CAM4-11-3854-s001.docx]

**Supplemental material**

**Semi-structured Interview Guide**

PREVENTATIVE CARE USE

1. How often do you see a doctor or another type of healthcare provider like a nurse practitioner or physician’s assistant?
2. What type of medical care do you get from these providers/doctors?
3. Do you see a doctor or provider for preventive care, meaning the type of care you get when you are not sick, but getting care can help prevent an illness or medical problem? For example, getting vaccine/immunization or test for heart problems or for cancer?
4. Do you usually see these providers in a hospital, or a medical office, or somewhere else?

REASONS FOR SCREENING AND BENEFIT VS HARM BALANCE

1. Now let’s talk about mammograms, the test that you have received in the past to check for breast cancer. Can you tell me why you get mammograms? (Probe: What motivates you to get a mammogram?
2. What do you think are some upsides (positives) and downsides (negatives) of getting mammograms? How would you compare the upsides of getting a mammogram to its downsides? (Probe: Are there more upsides than downsides? Are upsides more important than downsides or vice versa?)

POSITIVE VS NEGATIVE EXPERIENCES

1. In general, how would you describe your experiences of getting mammograms? How do you feel before, during and after you get a mammogram? (NOTE TO INTERVIEWER: A) if participant has already discussed her mammography experiences as part of question 3, skip this question; B) if participant has a hard time answering this question, direct her to think about her last mammogram).

EXPERIENCES WITH RECALL AND TESTING

1. Have you ever had to come back after having a mammogram to follow up with more mammograms or with other tests? Usually this happens to check something they may find on your screening mammogram. Please tell me more about these instances. How often/many times did this happen? Did you have to get more tests like more mammograms, breast ultrasound or MRI? Did they ever take a sample of your breast (this is called a biopsy)? How did you feel about having to go through these additional tests?

PROCESS OF MAMMOGRAM SCHEDULE

1. Now, please think about the last time you had a mammogram. Can you take me through the process of how you came to get your mammogram? For example, which doctor or healthcare provider usually refers you for a mammogram (PROBE: is it your primary care doctor, internist, etc)? How do you make your appointment? Do you usually call yourself to make your mammogram appointments or does your doctor’s office make them? Did you get a letter or reminder card to make your appointments? Did you get a phone call reminder? Text or email?
2. Does any of your family members of your friends help you with your mammogram appointments? For example, does anyone help you make your mammogram appointments or go with you to your mammogram? Tell me more about how s/he help you with your mammograms (PROBE: what is the relationship of this family member or friend).

SHARED DECISION MAKING

1. How do you make the decision to get a mammogram? (NOTE TO INTERVIEWER: allow participant to tell you how she makes the decision and then follow up with the following questions if not offered) Do you make the decision by yourself? Do you feel that your decision is influenced by your doctor (which type of doctor)? Family? Friends? Anyone else? (Probe for type/details of family member and friend)
2. Do you feel that you could use more discussion or information about mammograms? How satisfied are you with the information you have about mammograms? Tell me what you think would help you make better decisions about getting mammogram.
3. If you were unsure about getting a mammogram, whose advice or recommendation would influence your decision about whether or not to get a mammogram? PROBE: doctor (which doctor)? Family (which family member)? Friend (any specific friend? Describe this friend? (e.g., friend your age?)

HEALTHCARE OVERUSE

1. Some people think that some doctors or other healthcare providers recommend too many tests or treatment for patients. This is sometimes considered to be excessive healthcare or even unnecessary care. This issue sometimes comes up especially in the context of screening tests. Screening tests are tests that look for a possible disease of medical condition in patients who do not have any specific signs or symptoms from that disease. For example, screening mammograms are tests that look for breast cancer in women who do not have any breast problems. Have you ever heard or thought about the issue of getting too much healthcare? Please tell me about your thoughts. PROBE: [IF UNFAMILIAR], what do the terms “unnecessary or excessive care” mean to you? Please tell me what they mean in your own words.
2. In your opinion, what are some of the reasons or motivations for doctors to order too many screening tests or medical services?
3. Other than doctor’s recommendations, are there other reasons that you think contribute to patients receiving too much screening or medical services?

Scenario 1: Maria is 78 years old. She has diabetes or high blood sugar. She takes medication for her condition and has to see her doctor regularly. She has some difficulty handling routine everyday chores and needs help from her family and friends. She does not have a family history of breast cancer and has no signs of a breast problem such as a lump or any pain in her breast. Maria’s doctor does not think she needs to have a mammogram because of her age and her medical conditions, but leave the choice up to Maria to decide. What do you think Maria should do? Why?

Scenario 2: Christina is 78 years old. Overall her general health is good. She maintains a healthy lifestyle and even helps take care of her 5 grandchildren. She does not have a family history of breast cancer and she does not have any signs of a breast problem such as a lump or any pain in her breast. Christina’s doctor does not think she needs to have a mammogram because of her age, but leave the choice up to Christina to decide. What do you think Christina should do? Why?

**Codebook**

| **Code** | **Sub code** | **Definition** |
| --- | --- | --- |
| Personal reasons for mammogram.  Definition: Intrapersonal reasons for getting a mammogram and does not include direct interpersonal influences from doctors, family, or friends. | Routine/habit | Any mention of mammograms being something that they’ve always done, being routine, or a habit. |
|  | Previous connection family, friend | Any mention of having a personal connection to a family member or friend with breast cancer. Examples could be knowing women their age recently diagnosed with cancer. |
|  | History of diagnostic testing | Any mention of having to return for another mammogram, MRI, US, or biopsy following an initial mammography. |
|  | Proactive and preventive | Any mention of getting a mammography to detect cancer early or mammography’s being good for one’s overall health. |
|  | Covered by insurance | Any mention of health insurance covering the cost of a mammogram. Ex. Insurance covers it so why not do it. |
|  | Ease to do | Any mention of how getting a mammogram is easy so why not do it. |
|  | Peace of mind | Any mention of the reason for a mammogram is to put one’s mind at ease. |
|  | Symptomatic | Any mention of having breast related symptoms as a reason to get a mammogram. |
| Mammo attitudes and beliefs | Positives around screening | Responses to the advantages/positives of getting a mammogram. Ex. Makes one feel good, beneficial to overall health, staff making it a positive process, and early detection. |
|  | Negatives around screening | Responses to the disadvantages/negatives around screening including a specific reference to an emotion associated with the process or a negative experience. Ex. Radiation, pain, stress, and worry. |
|  | Overall balance of pros/cons | Direct responses to the question “how would you compare advantages/positives of getting a mammogram to its disadvantages/negatives. |
| Mammo behaviors | Frequency | Responses to how often the participants received a mammogram. |
|  | Age of initiation | Any mention of the age that one started to get mammograms or believes women should start getting mammograms |
|  | Age to stop | Any mention of a specific age to stop mammograms. Does not include the belief that women should never stop. |
| Mammo prompt reminder | Self/family reminder | Any mention of the participant reminding one’s self to get a mammogram or receiving reminders from family. |
|  | Doctor reminder or referral/during appointment | Any mention of their doctor (primary care/gyno/etc.) providing the participant with a referral or inquiring about a mammogram during an in-person visit. |
|  | Letter or call from insurance | Any communication from the insurance company reminding the participant that it is time to receive a mammogram. |
|  | Letter or call from clinic/hospital | Any communication from the screening clinic or facility reminding the participant that it is time to receive a mammogram. |
| Sources of information outside the physician | Family/friends | Any specific mention of going to family or friends for answers to health related questions. |
|  | Written or internet sources | Any specific mention of looking up information online or reading written materials from their doctor or clinic. |
|  | Satisfaction with information received | Direct response to satisfaction with the information one has received around mammograms. |
| Mammo decision influences  Definition: Interpersonal reasons for getting a mammogram. Does not include personal reasons or motivations to get a mammogram. | Doctor | Any mention of how a doctors recommendation or referral influences their decision to get a mammogram. |
|  | Family | Any mention of how family members encourage or recommend the participant to get a mammogram. |
|  | Friends/peers | Any mention of how friends/peers members encourage or recommend the participant to get a mammogram. |
| Perceptions of excessive/unnecessary care* | Reasons for overuse* | Any mention of reasons or causes for overuse. Ex. Doctors afraid of missing something, patient nonadherence, push from insurance companies. |
|  | Not aware | Response to not being aware of or disagreement about the existence of excessive or unnecessary care. Ex. All healthcare is necessary. |
|  | Aware | Response to being aware of or agreement that excessive or unnecessary care exists. |
|  | Experience with excessive care | Any personal experience with receiving unnecessary or excessive care. |
| Vignette response | Should get mammo no matter what | Any response to vignettes that the women should get a mammogram despite the doctor’s recommendation that it is not necessary. This should also capture their reasoning and my include age, number of chronic conditions, or the participants own experience. |
|  | Should follow doctor’s rec not to | Any response to vignettes that the women should follow the recommendation of their doctors. This should also capture their reasoning for following a doctor’s recommendation, such as trust, doing what the doctor says because they know best, or the participants own experience. |
|  | Needs more information from doctor | Any response to vignettes that the women should seek more information or a second opinion before making a decision. |
| Process of getting a mammogram* |  | Discussion of the process of getting a mammogram including from the time the participant receives a referral to the time they receive the results. |
| Overall Health/Service Utilization* |  | Discussion around overall health care use including the types of providers, number of visits, and reasons for visits not related to getting a mammogram. |

*Indicates codes/subcodes that may be expanded
